# Acupressure Potentially Promoting the Learning and Memory Capabilities of Hypoxic Ischemic Cerebral Palsy Rats

**DOI:** 10.1155/np/3901126

**Published:** 2026-08-28

**Authors:** Yiping Chen, Yongli Li, Yong Liao, Chenggui Xu, Xiantao Tai, Xinghe Zhang

**Affiliations:** ^1^ The Second Clinical Medical College, Yunnan University of Chinese Medicine, Kunming, Yunnan, China, ynutcm.edu.cn; ^2^ The First Clinical Medical College, Yunnan University of Chinese Medicine, Kunming, Yunnan, China, ynutcm.edu.cn

**Keywords:** acupressure, cerebral palsy, hippocampus, hypoxic ischemic, neuroplasticity

## Abstract

**Purpose:**

Whether acupressure could improve the learning and memory capabilities in hypoxic‐ischemic cerebral palsy (HIE) in rats and whether the mechanism is related to hippocampal neuroplasticity were investigated.

**Methods:**

In this study, we used a unilateral HIE rat model. There were three groups, including the sham control group, the HIE control group, and the acupressure group. The acupressure group was given a 4‐week treatment after the HIE modeling. The learning and memory ability of rats was tested by the open field, novel object recognition (NOR), Y maze, and Morris water maze (MWM) behavioral experiments. The hippocampal spine density and dendrites’ branch number and length were detected using Golgi staining. The thickness, width, and length of pre‐ and postsynaptic membranes were observed using electron microscopy. The postsynaptic α‐amino‐3‐hydroxy‐5‐methyl‐4‐isoxazolepropionic acid receptor (AMPAR) subunits AMPAR1, AMPAR2, and N‐methyl‐D‐aspartate receptor 2B (NMDAR2B) were visualized by immunohistochemistry and immunofluorescence staining. The brain neuron electrical activity at the rat hippocampal CA1 region was recorded by awake electroencephalography (EEG). Furthermore, the AMPAR and N‐methyl‐D‐aspartate receptor (NMDAR) protein expression in the hippocampus was detected using western blot.

**Results:**

Acupressure could alleviate HIE‐induced behaviors and promote the recovery of neural plasticity functions in the hippocampus, showing the broading of the synaptic cleft, improving the synaptic membrane, increasing dendrites’ branch numbers, prolonging the length of the dendrites, and enhancing the spine density (*p* < 0.05). The neural plasticity–related proteins in the hippocampus, including AMPAR and NMDAR, were all downregulated in the HIE group (*p* < 0.05). Immunofluorescence staining results displayed the same trend in terms of the NMDAR and AMPAR positivity rate (*p* < 0.05). However, these effects could be partly reversed after being treated by acupressure (*p* < 0.05). As for the EEG results, the spectrum became slow‐wave (increased δ and decreased θ) with disordered amplitudes in the model group, reflecting neuronal damage, while the spectrum partially recovered (θ rebounded and δ decreased) in the treatment group, suggesting the effectiveness of the intervention.

**Conclusion:**

Acupressure can ameliorate the learning and memory function of HIE by regulating the mechanism related to postsynaptic membrane, promoting the recovery of functional plasticity and neural plasticity–related electric signal upregulation in the hippocampus.

## 1. Introduction

Hypoxic‐ischemic cerebral palsy (HIE) events are significant contributors to long‐term neurological dysfunction in children, manifesting as dyskinesia, growth restriction, and cognitive development decline [1, 2]. HIE can also result in severe disability or mortality [3]. The primary cause of HIE is often associated with prematurity [4]. Nearly half of all deaths among children under the age of five occur within the first month of life, predominantly due to premature birth and complications during delivery [5]. According to reports by the World Health Organization (WHO), by 2030, an estimated 30 million children are projected to die before reaching the age of five [6]. Therefore, effective treatment and timely intervention for patients exposed to hypoxic‐ischemic episodes are imperative.

Existing research indicates that hypoxia and ischemia can impair neural plasticity in specific brain regions, and HIE has been found to be closely associated with alterations in neural plasticity [7]. As a significant component of modern medicine, clinical research has demonstrated that acupressure can enhance brain function and neurocognitive performance, thereby alleviating symptoms associated with HIE [8]. Numerous studies have substantiated the reparative effects of acupressure on neural plasticity [9, 10]. Furthermore, acupoints, particularly acupoint pressing therapy, have been clinically implemented in several countries for the treatment of patients with HIE, resulting in significant improvements in motor abilities and developmental outcomes [11, 12]. Additionally, the integration of acupoint stimulation with rehabilitation therapy is more readily accepted by patients, offers a safer approach, and yields superior therapeutic outcomes compared to rehabilitation therapy alone [13, 14]. Nevertheless, previous studies mainly focused on clinical or behavioral outcomes. The underlying mechanism of acupressure in improving learning and memory remains largely unclear.

Hypoxic‐ischemic injury can lead to learning and memory impairment, which is intricately linked to hippocampal neural plasticity, characterized by dynamic modifications in several critical proteins [15, 16]. Empirical studies have indicated that hypoxia‐ischemia inhibits the expression of postsynaptic density protein 95 (PSD95) and consequently disrupts N‐methyl‐D‐aspartate receptor (NMDAR) signaling complexes and compromises synaptic architecture [17, 18]. Particularly, cognitive impairment can be seen in hippocampal synaptic abnormalities [19]. The hippocampal CA1 region is critical for learning and memory, as it is the primary site of synaptic plasticity, for example, long‐term potentiation (LTP), and receives major cholinergic and glutamatergic inputs. Moreover, strain‐specific differences in synaptic plasticity are well documented in the Schaffer collateral–CA1 pathway, making it an ideal and reproducible target for electrophysiological and pharmacological studies of cognitive function [20]. Earlier electrophysiological recordings also show that postsynaptic NMDAR and the postsynaptic α‐amino‐3‐hydroxy‐5‐methyl‐4‐isoxazolepropionic acid receptor (AMPAR) play an important role in hippocampal synaptic plasticity [21]. Additionally, irregular activity of both NMDAR and AMPAR may be a cause of different types of central nervous system, including memory and behavioral disorders and cognitive defects [22]. Although acupressure therapy has been demonstrated to modulate neural plasticity and ameliorate hypoxic‐ischemic symptoms, its specific effects and associations with proteins related to hippocampal plasticity remain insufficiently elucidated. In particular, few studies have explored the relationship between acupressure and hippocampal synaptic plasticity at molecular, morphological, and electrophysiological levels, including AMPAR/NMDAR expression, dendritic remodeling, synaptic ultrastructure, and hippocampal awake electroencephalography (EEG)/local field potential (LFP) changes in HIE models.

Therefore, this study aims to determine whether acupressure at bladder meridian 23 (BL23, Shenshu) can improve learning and memory in HIE rats and to explore the underlying mechanism associated with hippocampal CA1 synaptic plasticity, including AMPAR/NMDAR expression, dendritic morphology, synaptic ultrastructure, and electrophysiological activity.

## 2. Materials and Methods

### 2.1. Animal Welfare

All experiments were performed in accordance with the Animal Research: Reporting of In Vivo Experiments (ARRIVE) guidelines, approved by the Animal Experimental Ethics Committee of Yunnan University of Chinese Medicine, and complied with the National Institutes of Health Guidelines for the Care and Use of Laboratory Animals (Ethics Serial Number R‐062023G145). The euthanasia procedures were consistent with the American Veterinary Medical Association (AVMA) Guidelines for the Euthanasia of Animals.

### 2.2. Experimental Design and HIE Procedure

**Figure 1:**
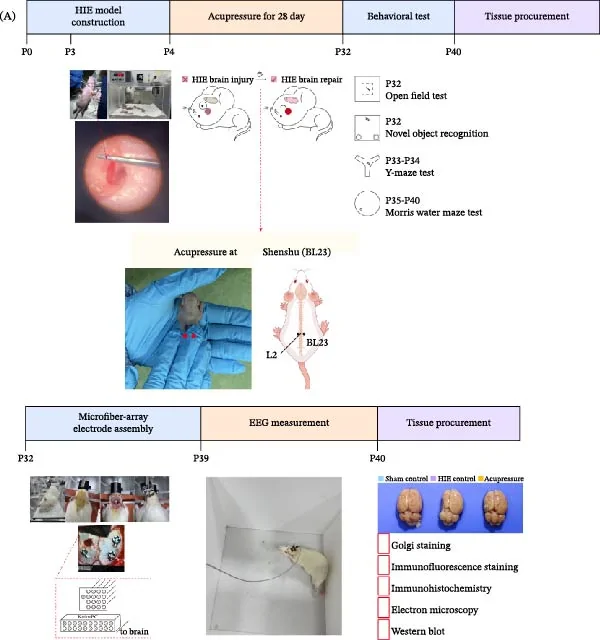
The technology roadmap of this paper: (A) The hypoxic‐ischemic models were construction on postnatal day 3 (P3). After mold making, acupressure was given for 28 days. Bilateral acupressure at BL23 points. Then, behavioral tests were conducted, and the microfiber‐array electrode was assembled on the hippocampus CA1. After recuperating for 1 week, EEG measurement was performed, and the tissues were harvested.

Four specific pathogen‐free (SPF) pregnant Sprague–Dawley rats, at gestational days 14–18 and weighing between 330–360 g, were procured and housed at the clean grade Laboratory Animal Center of Yunnan University of Chinese Medicine (License Number SCXK [JING] 2024‐0001). Following a period of acclimatization under controlled conditions (12‐h light/dark cycle, temperature 24 ± 2°C, and humidity 55 ± 15%), the offspring were kept in the same cage postdelivery. Based on existing literature, the hypoxic‐ischemic model is preliminarily established at 3 days postpartum [23]. On postnatal day 3 (P3), the pups (*n* = 30) were randomized across litters to three groups using a random number table: the sham control group (CN, *n* = 10), the HIE control group (HIE, *n* = 10), and the acupressure group (AP, *n* = 10). Before randomization, we first counted the number of P3 pups in each litter. The pups from the same litter were evenly distributed among the three groups as much as possible. This allocation strategy effectively controls for litter effects, ensures the statistical independence of each sample, and thereby guarantees the validity and reliability of the experimental results. The pups were anesthetized by induction with 2% isoflurane in an induction box, followed by maintenance anesthesia at 1% isoflurane delivered via a facial inhalation mask using a small‐animal gas anesthesia workstation. Both the HIE and AP groups underwent ischemia and hypoxia to induce the hypoxic‐ischemic model. The left common carotid artery was isolated from the carotid sheath through blunt dissection, cauterized using a bipolar electrocoagulator (Vetroson V‐10 B1‐Polar Electrosurgical Unit, Summit Hill Laboratories, Navesink, NJ), and the incision was sutured. A similar surgical procedure, excluding carotid artery ligation, was performed on the CN group. The rats’ behavior was monitored to assess their condition. The study roadmap is shown in Figure 1.

### 2.3. Acupressure Intervention

Based on the establishment of the initial model, we performed acupressure treatment for 4 weeks (Figure 1). A similar protocol was present in our previous study. Briefly, rats in the AP group were given acupressure treatment at BL23 daily. The parameters of acupressure are determined based on previous research (100–120 times/min, 2 min, and 1.77 ± 0.54 N) [24]. All individuals performing the acupressure were rigorously trained and assessed by a supervising professor prior to the experiment; only those who passed the qualification criteria participated in the study. The acupressure procedure was carried out with particular attention to maintaining the pups in a calm and comfortable state throughout. Additionally, during the acupressure intervention period, pups in the sham control group were housed separately from their dams to minimize potential experimental confounding variables.

### 2.4. Baseline and Behavioral Test

All behavioral assays, including the open field test (OFT), novel object recognition (NOR) test, Y‐maze test, and Morris water maze (MWM) test, were administered at the conclusion of the experimental period.

OFT and NOR were conducted within a square arena measuring 100 × 100 × 40 cm, featuring black walls and a black base. Each rat was permitted 5 min of unrestricted exploration. As spatial exploration demonstrates memory and the ability to learn, we used OFT to evaluate rats’ exploratory locomotor activity by measuring total distance traveled, average velocity, and immobility duration [25, 26].

The NOR assessed short‐term cognitive memory by leveraging the rodents’ inherent preference for novel stimuli [27]. Cognitively intact animals typically demonstrate significantly longer exploration times for novel objects, whereas cognitively impaired animals do not exhibit a preference. The recognition index (RI) was calculated using the formula: RI = (time spent exploring the novel object/total time spent exploring both objects) × 100%.

The Y‐maze test assessed spatial recognition memory using a Y‐shaped apparatus, with each arm measuring 55 × 15 cm [28]. Spontaneous alternation behavior, defined as consecutive entries into all three arms without repetition, was recorded to quantify the exploration of the novel arm.

The MWM assessed long‐term spatial learning and memory, which was evaluated using a circular pool (diameter: 130 cm) filled with black water (22 ± 2°C) colored with nontoxic dye [29, 30]. A hidden platform (diameter: 16 cm) was submerged 1 cm below the water surface and semirandomly positioned in the target quadrant (Quadrant III). Day 1 involved platform exposure in all quadrants to habituate rats to the task. Over 4 training days (Days 2–5), rats received four trials per day to locate the platform. On Day 5, a probe trial was conducted without the platform. Any‐Maze tracking software recorded escape latency, path efficiency, and swim speed.

### 2.5. EEG Data Acquisition and Processing

EEG signals were recorded using 16 NeuroWare neural signal acquisition system (ldeacentre). Rats were anesthetized with 2% isoflurane for induction, followed by maintenance anesthesia at 1.0%–1.5% isoflurane, and fixed to a stereotaxic frame. The rats’ heads were shaved, and the scalps were removed. A dental precision drilling machine was used to drill four holes in the skull. One hole was fitted with a microfilament array electrode to record EEG from the left hippocampus (AP: +3.0 mm, ML: ±3 mm, and DV: 3.6 mm), defined relative to the anterior fontanelle (Figure 1A). The other three holes were installed with micro screws (1 mm × 1.2 mm) to stabilize the electrodes. All EEG electrodes are 20 μm needle wires, 16 channels, 200 micron spacing micro wire array, and 5 mm axis length (Yunnan Sengge Biotechnology Co., Ltd., 20250212001, KedouBC), as shown in Figure 1A. During the experiment, the microfilament impedance of all electrodes was kept between 100 and 300 K. The electrodes are fixed to the skull with a light‐curing agent, and all wires are connected to micro screws. Next, dental cement (Stoelting) was applied around the head of the connection to protect all wires and connectors. All rats were allowed to recover in a separate cage postoperatively. The recording of brain activity for the hippocampus area CA1 was performed 1 week after the electrode implantation. Lastly, the LFP characteristics of the hippocampus area CA1 were recorded (sampling frequency: 2000 Hz and bandpass: 0.1–500 Hz).

### 2.6. Euthanasia Process

After behavioral assessments, rats were euthanized by inhalation of 5% isoflurane for deep anesthetic overdose, followed by rapid cervical dislocation to verify permanent death. Cessation of respiration and corneal reflex for more than 3 min were recorded before brain tissue dissection.

### 2.7. Golgi Staining

Golgi staining for hippocampal dendritic spine density and morphology was performed following the FD Rapid Golgi Stain kit protocol (FD NeuroTechnologies, PK401). Brains of SD rats were immersed in the impregnation solution (solution A mixed with an equal volume of B) for 14 days and avoid light at room temperature. The number of nerve cells, dendrites’ length, and spine density in the hippocampus CA1 and CA3 areas were observed and calculated through Fiji ImageJ software.

### 2.8. Electron Microscopy Observation

1 mm^3^ in volume sections of the hippocampus area CA1 was fixed in 2.5% glutaraldehyde and postfixated in 1% osmic acid. The parameters for dehydration, embedding, and polymerization of the specimens are as follows: 10 min in 30% ethanol, 10 min in 50% ethanol, 15 min in 70% ethanol, 15 min in 90% ethanol, 15 min in anhydrous ethanol, 15 min in acetone (twice), 30 min in a resin‐acetone 1:3 mixture, 30 min in a resin‐acetone 1:1 mixture, 1 h in a resin‐acetone 3:1 mixture, 37°C overnight in the resin embedding solution, 40°C of new embedding solution, immersion for 8 h, and 60°C polymerization for 48 h. The enameled blocks were cut into sections with a thickness of 0.8 μm, stained with toluidine blue, and observed under light microscope to locate the areas where astrocytes were relatively dense. The slices with a thickness of 90 nm were pasted on the square hua membrane copper mesh with a mesh size of 200. Conventional uranium acetate–lead citrate electron staining was performed for 15 min and 5 min, respectively, and dried after staining. The ultrathin section holder was then imaged and captured at 80 kV on the Hitachi HT7800 transmission electron microscope. The synaptic action zone length, synaptic cleft width, and postsynaptic density (PSD) thickness in the hippocampus CA1 and CA3 areas were observed and calculated through Fiji ImageJ software.

### 2.9. Immunofluorescence Staining

Postsynaptic NMDAR and AMPAR were visualized by immunofluorescence staining. The brain, fixed with polyformaldehyde, was embedded in paraffin and cut into 10 μm thick sections. After dewaxing and hydration, the antigen was repaired, and primary antibodies were incubated overnight at 4°C in a 5% SNIPER blocking solution, with concentrations as follows: monoclonal rabbit anti‐NMDAR2B (1:200), polyclonal rabbit anti‐AMPAR1 (1:200), and monoclonal rabbit anti‐AMPAR2 (1:70). After washing steps, secondary antibodies were incubated for 1 h at a dilution of 1:500 in a 5% SNIPER blocking solution (Lianke Bio, goat antirabbit Alexa Fluor 555 #A27039). Following three TBST washes, the slides were incubated for 7 min at a dilution of 1:500 in a 5% SNIPER solution containing ThermoFisher Scientific DAPI (Sailor, G1012) #D1306. Finally, the slides were sealed with an antifluorescence quencher mounting medium. The quantitative analyses were measured using ImageJ software.

### 2.10. Immunohistochemistry

The immunohistochemical staining was performed as previously described. Paraffin sections were dewaxed to water. After antigen repair and close endogenous peroxidase blockage, the sections were incubated with primary antibodies at 4°C overnight. The following day, sections were washed with PBS and stained with secondary antibodies, and diaminobenzidine coloration was performed subsequently. The quantitative analyses were then measured using ImageJ software.

### 2.11. Western Blot

The expression of AMPAR and NMDAR proteins in the hippocampus was detected using western blot. First, the total protein was extracted according to standard methods. Proteins were blocked with 5% skim milk for 2 h. The primary antibodies were incubated overnight. After that, the membranes were washed with PBS 3 × 5 min and incubated with secondary antibody for 1 h at room temperature. Next, proteins were separated by electrophoresis, transferred to a PVDF membrane, and finally exposed for signal detection using ECL substrate.

### 2.12. Statistical Analysis

All data were processed by GraphPad Prism 8.0 Windows software. Data were expressed as mean ± standard deviation (SD). For statistical analysis of multiple groups, one‐way analysis of variance (ANOVA) was applied. Mean differences (*MDs*) and corresponding 95% confidence intervals (*95% CIs*) in the outcomes were estimated. Statistical results were considered significant when *p*  < 0.05.

## 3. Results

### 3.1. Acupressure Attenuates the Manifestations of Cerebral Palsy in HIE‐Induced Rats

**Figure 2:**
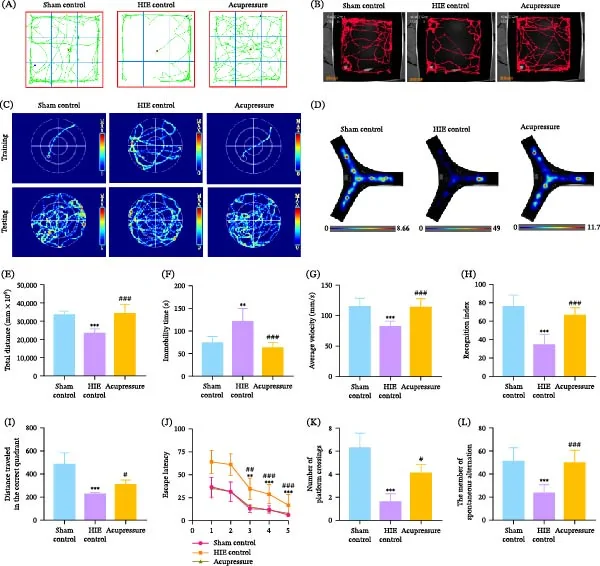
Animal behavioral test: (A) The green lines represent the trajectory of rats in OFT. (B) The red lines represent the trajectory of rats in the NOR test. (C) The training and testing traces of rats in the MWM. (D) The path traces of each group in the Y‐maze test. (E) Total distance of rats in each group in the OFT, *n* = 10. (F) The immobility time of rats in each group in OFT, *n* = 10. (G) The average velocity of rats in each group in the OFT, *n* = 10. (H) The recognition index of rats in each group in the NOR test, *n* = 10. (I) The distance traveled in the correct quadrant in the MWM, *n* = 10. (J) The escape latency of rats in each group, *n* = 10. (K) The number of platform crossing in the MWM, *n* = 10. (L) The number of spontaneous alternation in each group in Y‐maze test, *n* = 10. Abbreviations: MWM, Morris water maze test; NOR, novel object recognition; OFT, open field test. Compared with the sham control group,  ^∗∗^ *p* < 0.01,  ^∗∗∗^ *p* < 0.001; compared with the HIE control group, ^#^ *p* < 0.05, ^##^ *p* < 0.01, ^###^ *p* < 0.001.

The behavioral results are shown in Figure 2 and Table 1. The OFT results suggested decreased exercise capacity in HIE control rats when compared with rats in the sham control group (total distance: *MD* = −10084, *95% CI*: −12,747 to −7420, *p*  < 0.001; immobility time: *MD* = −46.24, *95% CI*: −73.45 to −19.02, *p*  < 0.01; average velocity: *MD* = −32.99, *95% CI*: −47.02 to −18.95, *p*  < 0.001). After 4 weeks of treatment, motor ability of rats in the acupressure group was increased markedly compared to the HIE control group (total distance: *MD* = 10936, *95% CI*: 6276–15596, *p*  < 0.001; immobility time: *MD* = 57.60, *95% CI*: 30.75–84.46, *p*  < 0.001; average velocity: *MD* = 31.73, *95% CI*: 17.69–45.76, *p*  < 0.001). To observe the effects of acupressure on the cognition of the rats, the NOR, MWM, and Y‐maze tests were conducted. The rats in the HIE control group spent shorter time on the novel object compared to the sham control group (*MD* = −41.33, *95% CI*: −53.24 to −29.42, *p*  < 0.001). When compared with the HIE control group, the NOR index of rats in the acupressure group increased (*MD* = 31.84, *95% CI*: 19.92–43.75, *p*  < 0.05); meanwhile, rats in acupressure group and sham control group showed similar preference for the novel object (*MD* = 9.496, *95% CI*: −2.416 to 21.41, *p*  > 0.05). In the MWM test, compared with the sham control group, the HIE control group exhibited significantly shorter distance traveled in the correct quadrant (*Kruskal–Wallis statistic* = 25.81, *p*  < 0.001), prolonged escape latency (*MD* = −21.30, *95% CI*: −26.57 to −16.03, *p*  < 0.001), and decreased number of platform crossings (*Kruskal–Wallis statistic* = 25.38, *p*  < 0.001). After acupressure intervention, the escape latency was markedly reduced (*MD* = 20.83, *95% CI*: 16.25–25.40, *p*  < 0.05), while the number of platform crossings (*Kruskal–Wallis statistic* = 25.38, *p*  < 0.05) and the distance in the target quadrant (*Kruskal–Wallis statistic* = 25.81, *p*  < 0.001) were both significantly increased compared with the HIE control group.

**Table 1:** Animal behavioral test (mean ± SD).

Group		Sham control (*n* = 10)	HIE control (*n* = 10)	Acupressure (*n* = 10)
Total distance		33,347 ± 2152	23,263 ± 2495 ^∗∗∗^	34,199 ± 5005^###^
Immobility time		74.12 ± 13.47	120.4 ± 29.54 ^∗∗^	62.75 ± 11.57^###^
Average velocity		114.9 ± 13.92	81.90 ± 9.25 ^∗∗∗^	113.6 ± 14.19^###^
Recognition index		75.85 ± 12.54	34.52 ± 10.82 ^∗∗∗^	66.36 ± 8.48^###^
Distance traveled in the correct quadrant		486.4 ± 97.50	226.5 ± 15.61 ^∗∗∗^	307.8 ± 40.75^#^
Escape latency				
Day 1		36.53 ± 10.71	64.07 ± 12.51	35.46 ± 11.42
Day 2		31.52 ± 10.65	61.06 ± 11.77	31.84 ± 8.60
Day 3		13.01 ± 4.05	34.66 ± 11.68 ^∗∗^	15.03 ± 3.09^###^
Day 4		11.99 ± 3.93	29.02 ± 10.40 ^∗∗∗^	11.31 ± 3.31^###^
Day 5		6.1 ± 1.46	16.83 ± 11.73 ^∗∗∗^	7.87 ± 3.05^###^
Number of platform crossing		6.30 ± 1.25	1.60 ± 0.69 ^∗∗∗^	4.10 ± 0.74^#^
Number of spontaneous alternation		50.83 ± 12.08	23.50 ± 7.25 ^∗∗∗^	49.61 ± 10.96^###^

*Note:* Compared with the sham control group,  ^∗∗^ *p* < 0.01 and  ^∗∗∗^ *p* < 0.001; compared with the HIE control group, ^#^ *p* < 0.05 and ^###^ *p* < 0.001.

### 3.2. Acupressure Repairs the Neural Features of HIE‐Induced Rats

**Figure 3:**
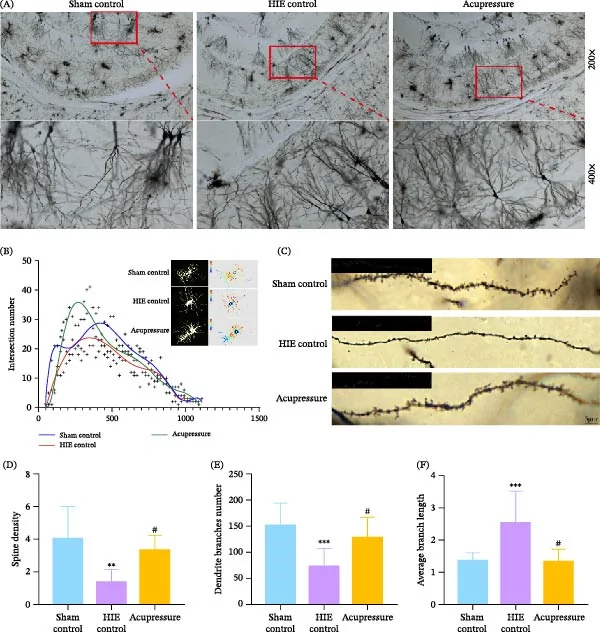
Effects of acupressure on synaptic spine density and synaptic ultrastructure in hippocampal CA1 neurons: (A) Representative images of neurons stained with the Golgi method in each group. (B) Number of intersection and concentric circles in hippocampal slices in each group. (C) Representative images of dendritic arborization in each group. (D–F) Quantitative analysis of the spine density, dendrite branches number, and average branch length in the hippocampus of each group rats, *n* = 6. Compared with the sham control group,  ^∗∗^ *p* < 0.01,  ^∗∗∗^ *p* < 0.001; compared with the HIE control group, ^#^ *p* < 0.05.

To visualize the neuronal morphology in detail, we used Golgi staining. The staining images showed that the hippocampal neuronal cells in the HIE control group were relatively rare and fragmented, represent as disorganized synaptic morphology and lower dendritic spine density, compared to the sham control group (spine density: *MD* = 2.679, *95% CI*: 1.204–4.155, *p*  < 0.01; dendrites branches number: *MD* = 78.33, *95% CI*: 34.69–122.0, *p*  < 0.001; average branch length: *MD* = −1.155, *95% CI*: −2.072 to −0.2386, *p*  < 0.001). While all the features of neurons were partly recovered in acupressure group compared to the HIE control group (spine density: *MD* = −1.946, *95% CI*: −3.422 to −0.4705, *p*  < 0.05; dendrites branches number: *MD* = 1.193, *95% CI*: 0.2626–2.124, *p*  < 0.05; average branch length: *MD* = −55.78, *95% CI*: −99.42 to −12.14, *p*  < 0.05) (see Figure 3 and Table 2).

**Table 2:** Effects of acupressure on synaptic spine density and synaptic ultrastructure in hippocampal CA1 neurons (mean ± SD).

Group	Sham control (*n* = 6)	HIE control (*n* = 6)	Acupressure (*n* = 6)
Spine density	4.13 ± 1.88	1.45 ± 0.70 ^∗∗^	3.39 ± 0.84^#^
Dendrite branch number	153.80 ± 41.68	75.44 ± 32.83 ^∗∗∗^	131.20 ± 36.17^#^
Average branch length	1.41 ± 0.19	2.57 ± 0.96 ^∗∗∗^	1.37 ± 0.35^#^

*Note:* Compared with the sham control group,  ^∗∗^ *p* < 0.01 and  ^∗∗∗^ *p* < 0.001; compared with the HIE control group, ^#^ *p* < 0.05.

**Figure 4:**
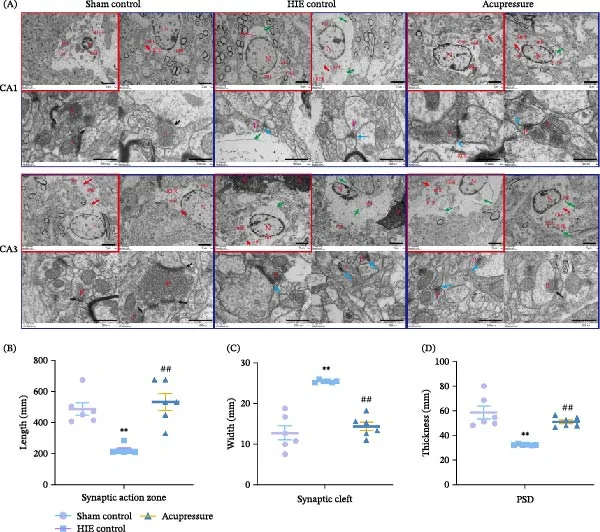
The effect of acupressure on astrocytes and synapses: (A) Electron microscopy representative images of rat astrocytes and synapses among groups. Scale bars: 50 μm and 20 μm as indicated in the images. (B) The synaptic action zone length in each group, *n* = 6. (C) The synaptic cleft width in each group, *n* = 6. (D) The postsynaptic density thickness in each group, *n* = 6. Abbreviations: black→, synaptic clefts; blue→, abnormal synaptic clefts; Gol, Golgi complex; green→, membrane fragments from damaged or autophagotic structures; mit, mitochondrion; N, glial cell nucleus; P, presynaptic components and vesicles; red→, glial fibrils; RER, rough endoplasmic reticulum. Compared with the sham control group,  ^∗∗^ *p* < 0.01; compared with the HIE control group, ^##^ *p* < 0.01.

Further analyses on neuron integrity were performed with the help of electron microscopy. The ultrastructure of the neural tissue in CA1 and CA3 regions of the rat hippocampus was observed, focusing on astrocytes and their surrounding synapses. In this experiment, the astrocytes in CA1 and CA3 of the sham control group showed normal ultrastructure with their surrounding synapses. The most significant changes in the HIE control group included swelling of glial cell bodies, disordered or damaged glial fibrils, reduced cytoplasmic electron density, and the presence of membrane‐fragmented structures or autophagosomes within the cytoplasm. Glial cell nuclei showed fewer abnormalities, and mitochondrial abnormalities were rare. The primary changes in the synaptic structure were a general narrowing of functional contact surfaces and a scarcity of vesicles in the presynaptic component. In particular, the damage to glial cells in the CA3 region was more severe, with noticeable swelling of glial cell protrusions around the microvessels. In the acupressure groups, glial cells also exhibited varying degrees of swelling and intracellular autophagosomes, but the visibility of glial fibrils was higher, and the rate of synaptic abnormalities was lower compared to the HIE control group. The presynaptic membrane, postsynaptic membrane, and synaptic cleft of electron micrographs in each group were clear and quantified. After modeled, the synapses of neurons in the hippocampus of HIE rats significantly weakened by comparing of rats in sham control group, including shorted synaptic action zone length (*MD* = 262.2, *95% CI*: 133.0–391.3, *p*  < 0.01), widened synaptic cleft width (*MD* = −12.69, *95% CI*: −17.00 to −8.389, *p*  < 0.01), and pinched PSD thickness (*MD* = 26.16, *95% CI*: 9.153–43.17, *p*  < 0.01). In contrast, acupressure treatment remarkably reversed these in the hippocampus of HIE control rats (length: *MD* = −307.0, *95% CI*: −482.0 to −132.0, *p*  < 0.01; width: *MD* = 11.05, *95% CI*: 6.745–15.35, *p*  < 0.01; PSD: *MD* = −18.55, *95% CI*: −23.83 to −13.27, *p*  < 0.01) (see Figure 4 and Table 3).

**Table 3:** The effect of acupressure on astrocytes and synapses (mean ± SD).

Group	Sham control (*n* = 6)	HIE control (*n* = 6)	Acupressure (*n* = 6)
Synaptic action zone length	490.20 ± 98.22	228.10 ± 29.25 ^∗∗^	535.10 ± 132.70^##^
Synaptic cleft width	12.79 ± 4.26	25.48 ± 0.28 ^∗∗^	14.43 ± 2.56^##^
Postsynaptic density thickness	58.91 ± 12.81	32.75 ± 0.57 ^∗∗^	51.30 ± 3.99^##^

*Note:* Compared with the sham control group,  ^∗∗^ *p* < 0.01; compared with the HIE control group, ^##^ *p* < 0.01.

### 3.3. Acupressure Alleviates Overactivity of the Hippocampal NMDAR/AMPAR Axis in HIE‐Induced Rats

**Figure 5:**
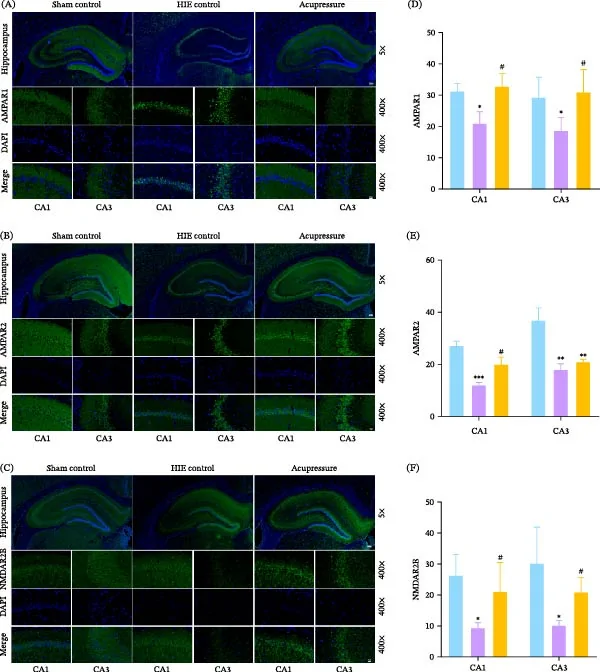
Effects of acupressure on neuronal postsynaptic AMPA‐ and NMDA‐subtype protein expressions in hippocampal CA1 and CA3 neurons: (A–C) Representative images of AMPAR1, AMPAR2, and NMDAR2B in each group. (D–F) Relative fluorescence intensity of AMPAR1, AMPAR2, and NMDAR2B in hippocampal CA1 and CA3, *n* = 3. Compared with the sham control group,  ^∗^ *p* < 0.05,  ^∗∗^ *p* < 0.01,  ^∗∗∗^ *p* < 0.001; compared with the HIE control group, ^#^ *p* < 0.05.

**Figure 6:**
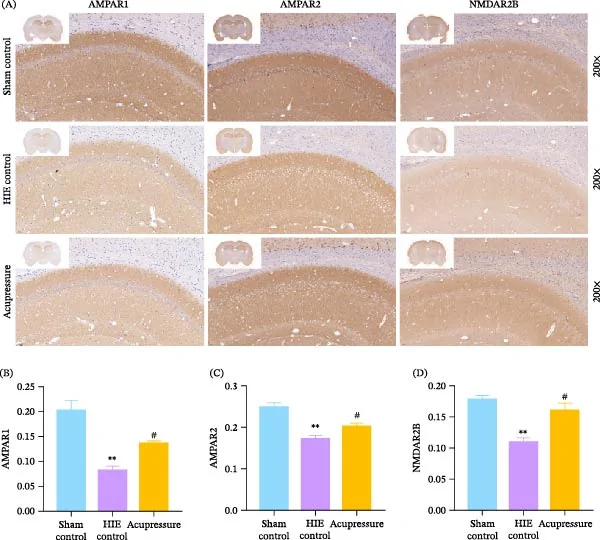
Effects of acupressure on hippocampal expression of neuronal postsynaptic AMPA and NMDA receptors: (A) Representative images of NMDAR and AMPAR subtype protein immunohistochemical staining. (B) Quantification of relative AMPAR1 expression, *n* = 3. (C) Quantification of relative AMPAR2 expression, *n* = 3. (D) Quantification of relative NMDAR2B expression, *n* = 3. Compared with the sham control group,  ^∗∗^ *p* < 0.01; compared with the HIE control group, ^#^ *p* < 0.05.

Immunohistochemistry and immunofluorescence staining were applied to check the the expression of hippocampal NMDA and AMPA subunit receptors, which play an important role in object recognition memory. As shown in Figures 5 and 6 and Tables 4 and 5, the expression levels of AMPAR1, AMPAR2, and NMDAR2B were significantly downregulated in the HIE control group (immunofluorescence: AMPAR1 [CA1: *MD* = 10.35, *95% CI*: 1.407–19.29, *p*  < 0.05; CA3: *MD* = 11.10, *Kruskal–Wallis statistic* = 5.422, *p*  < 0.05], AMPAR2 [CA1: *MD* = 15.32, *95% CI*: 10.49–20.15, *p*  < 0.001; CA3: *MD* = 18.86, *95% CI*: 8.64–43.51, *p*  < 0.01], NMDAR2B [CA1: *MD* = 16.62, *95% CI*: 5.02–28.21, *p*  < 0.05; CA3: *MD* = 15.44, *95% CI*: 1.227–38.83, *p*  < 0.05]; immunohistochemistry: AMPAR1: *MD* = 0.1213, 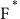 = 88.21, *p*  < 0.01; AMPAR2: *MD* = 0.07709, *95% CI*: 0.06075–0.09343, *p*  < 0.01; NMDAR2B: *MD* = 0.06892, *95% CI*: 0.05105–0.08679, *p*  < 0.01); however, these effects could be partly reversed after treatment by acupressure (immunofluorescence: AMPAR1 [CA1: *MD* = −11.82, *95% CI*: −20.76 to −2.879, *p*  < 0.05; CA3: *MD* = −12.80, *Kruskal–Wallis statistic* = 5.422, *p*  < 0.05], AMPAR2 [CA1: *MD* = −7.862, *95% CI*: −12.69 to −3.03, *p*  < 0.05; CA3: *MD* = −3.036, *95% CI*: −12.00 to 5.931, *p*  > 0.05], NMDAR2B [CA1: *MD* = −11.33, *95% CI*: 5.093–13.83, *p*  < 0.05; CA3: *MD* = −10.66, *95% CI*: −29.46 to 8.137, *p*  < 0.05]; immunohistochemistry: AMPAR1: *MD* = −0.05503, 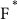 = 88.21, *p*  < 0.05; AMPAR2: *MD* = −0.03033, *95% CI*: −0.04667 to −0.01399, *p*  < 0.05; NMDAR2B: *MD* = −0.05094, *95% CI*: −0.06881 to −0.03307, *p*  < 0.05).

**Table 4:** Effects of acupressure on neuronal postsynaptic AMPA‐ and NMDA‐subtype protein expressions in hippocampal CA1 and CA3 neurons (mean ± SD).

Group Hippocampal region		Sham control (*n* = 3)	HIE control (*n* = 3)	Acupressure (*n* = 3)
AMPAR1	CA1	31.41 ± 2.31	21.06 ± 3.87 ^∗^	32.88 ± 4.23^#^
	CA3	29.04 ± 6.81	17.94 ± 4.81 ^∗^	30.74 ± 7.6^#^
AMPAR2	CA1	26.80 ± 2.05	11.49 ± 1.45 ^∗∗∗^	19.35 ± 3.35^#^
	CA3	36.56 ± 5.52	17.70 ± 2.50 ^∗∗^	20.73 ± 1.31 ^∗∗^
NMDAR2B	CA1	26.08 ± 7.02	9.46 ± 1.76 ^∗^	20.79 ± 9.91^#^
	CA3	29.95 ± 11.80	9.923 ± 2.03 ^∗^	20.59 ± 5.06^#^

*Note:* Compared with the sham control group,  ^∗^ *p* < 0.05,  ^∗∗^ *p* < 0.01, and  ^∗∗∗^ *p* < 0.001; compared with the HIE control group, ^#^ *p* < 0.05.

**Table 5:** Effects of acupressure on hippocampal expression of neuronal postsynaptic AMPA and NMDA receptors (mean ± SD).

Group	Sham control (*n* = 3)	HIE control (*n* = 3)	Acupressure (*n* = 3)
AMPAR1	0.21 ± 0.02	0.08 ± 0.01 ^∗∗^	0.14 ± 0.002^#^
AMPAR2	0.25 ± 0.008	0.17 ± 0.006 ^∗∗^	0.20 ± 0.005^#^
NMDAR2B	0.18 ± 0.005	0.11 ± 0.005 ^∗∗^	0.16 ± 0.01^#^

*Note:* Compared with the sham control group,  ^∗∗^ *p* < 0.01; compared with the HIE control group, ^#^ *p* < 0.05.

### 3.4. Acupressure Attenuated the EEG of Hippocampal Neurals of HIE‐Induced Rats

**Figure 7:**
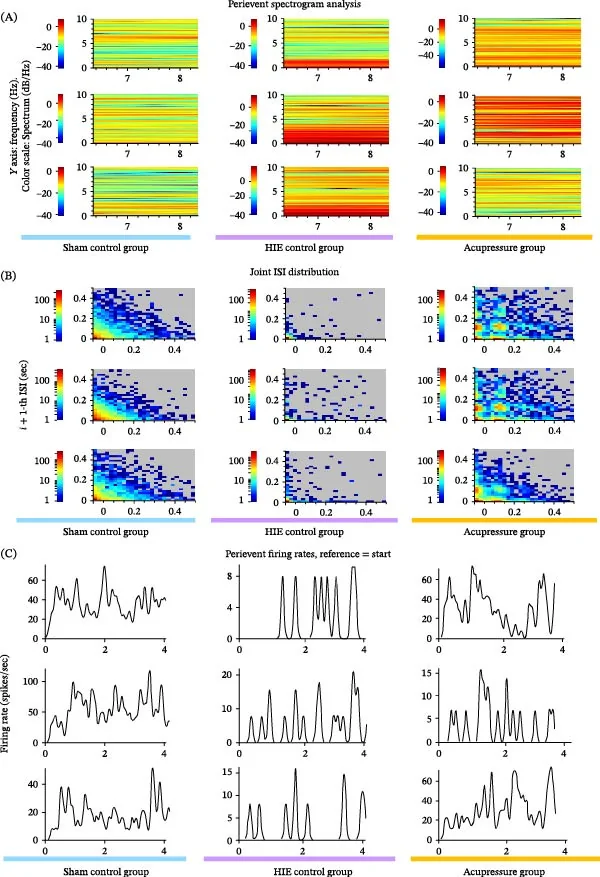
Effects of acupressure on hippocampal LFP: (A) Perievent spectrogram analysis in each group, *n* = 3. (B) Joint ISI distribution in each group, *n* = 3. (C) Perievent firing rates in each group, *n* = 3.

To fully exploit the relationship between NMDA/AMPA and behavioral learning and memory, we performed an exploratory single‐trial EEG‐informed hippocampus analysis. The spectral characteristics of the sham control group rats were primarily in the θ band (4–8 Hz), characterized by clear and concentrated peaks, reflecting normal hippocampal functions such as memory‐ and spatial navigation–related theta rhythms. The δ band (1–4 Hz) and the β/γ band (>13 Hz) had lower energy levels with no significant abnormal fluctuations. The overall spectral distribution was regular, with a stable baseline and no pathological waveforms, such as spikes or sharp waves. The joint interstimulus interval (ISI) distribution showed that the frequency distribution was concentrated in the θ band, presenting as a single peak or narrow distribution, with a clear relationship between power values (on the *y*‐axis) and frequencies (on the *x*‐axis). The amplitude distribution was uniform, with no extreme high‐amplitude events (such as epileptic discharges). After modeling, the energy in the θ band significantly decreased, and the main frequency may have shifted to the δ band (slow wave), indicating a decrease in neuronal excitability. The energy in the δ band (1–4 Hz) increased, accompanied by intermittent abnormal increases in the β/γ band (>13 Hz). The spectral baseline was unstable, with spikes, sharp waves, or paroxysmal high‐amplitude θ/δ waves. The frequency distribution was broad, with an increased proportion of the δ band and a weakened or absent peak in the θ band. The amplitude of perievent firing rates was skewed to the right with more high‐amplitude events and shows a bimodal distribution with a mix of normal and pathological waveforms. In the HIE control group, the spectrum became slow‐wave (increased δ and decreased θ), with disordered amplitudes, reflecting neuronal damage; in the acupressure group, the spectrum partially recovered (θ rebounded and δ decreased), suggesting the effectiveness of the intervention. In the acupressure group, the energy in the abnormal bands (δ and β/γ) decreased, with the amplitude distribution approaching normal but not fully restored to the sham control group level (see Figure 7).

### 3.5. Acupressure Increases the Expressions of Neural Postsynaptic Membrane–Related Proteins in the Hippocampus of HIE‐Induced Rats

**Figure 8:**
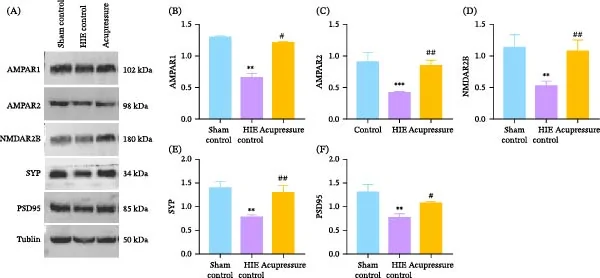
Effects of acupressure on hippocampal synaptic plasticity related protein: (A) The protein bands visualized by western blot, *n* = 3. (B) Protein expression of AMPAR1 in each group, *n* = 3. (C) Protein expression of AMPAR2 in each group, *n* = 3. (D) Protein expression of NMDAR2B in each group, *n* = 3. (E) Protein expression of SYP in each group, *n* = 3. (F) Protein expression of PSD95 in each group, *n* = 3. Compared with the sham control group,  ^∗∗^ *p* < 0.01,  ^∗∗∗^ *p* < 0.001; compared with the HIE control group, ^#^ *p* < 0.05, ^##^ *p* < 0.01.

Subsequently, we evaluated the effects of acupressure on the postsynaptic membrane‐related proteins, including AMPAR1, AMPAR2, NMDAR2B, synaptophysin (SYP), and PSD95, in the hippocampus. As shown in Figure 8 and Table 6, the expressions of AMPAR1, AMPAR2, NMDAR2B, SYP, and PSD95 were all downregulated in the HIE control group (AMPAR1: *MD* = 0.6424, *95% CI*: 0.5641–0.7206, *p*  < 0.01; AMPAR2: *MD* = 0.4875, *95% CI*: 0.2590–0.7159, *p*  < 0.001; NMDAR2B: *MD* = 0.6100, 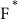 = 16.00, *p*  < 0.01; SYP: *MD* = 0.6193, *95% CI*: 0.3614–0.8771, *p*  < 0.01; PSD95: *MD* = 0.5408, *95% CI*: 0.3116–0.7700, *p*  < 0.01); however, these effects could be relatively reversed by treatment of acupressure (AMPAR1: *MD* = −0.5487, *95% CI*: −0.6270 to −0.4705, *p*  < 0.05; AMPAR2: *MD* = −0.4263, *95% CI*: −0.6548 to −0.1979, *p*  < 0.01; NMDAR2B: *MD* = −0.5516, 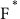 = 16.00, *p*  < 0.01; SYP: *MD* = −0.5210, *95% CI*: −0.7789 to −0.2632, *p*  < 0.01; PSD95: *MD* = −0.3052, *95% CI*: −0.5344 to −0.07601, *p*  < 0.05).

**Table 6:** Effects of acupressure on hippocampal synaptic plasticity–related protein (mean ± SD).

Group	Sham control (*n* = 3)	HIE control (*n* = 3)	Acupressure (*n* = 3)
AMPAR1	1.31 ± 0.002	0.67 ± 0.05 ^∗∗^	1.22 ± 0.005^#^
AMPAR2	0.92 ± 0.14	0.43 ± 0.01 ^∗∗∗^	0.86 ± 0.07^##^
NMDAR2B	1.15 ± 0.19	0.54 ± 0.06 ^∗∗^	1.09 ± 0.16^##^
SYP	1.42 ± 0.12	0.80 ± 0.03 ^∗∗^	1.317 ± 0.13^##^
PSD95	1.33 ± 0.14	0.79 ± 0.07 ^∗∗^	1.09 ± 0.02^#^

*Note:* Compared with the sham control group,  ^∗∗^ *p* < 0.01 and  ^∗∗∗^ *p* < 0.001; compared with the HIE control group, ^#^ *p* < 0.05 and ^##^ *p* < 0.01.

## 4. Discussion

HIE is a continuously evolving disease [31], with advancing weakness and spasticity [32]. Nowadays, available therapies have severe limitations including high toxicity and systemic side effects [33]. Recently, acupressure, a manual acupoint stimulation technique that involves pressing on the specific acupoints, has been suggested that can improve brain function and promote nerve development [34]. However, the underlying mechanism of acupressure in the HIE learning and memory treatment is still unclear. In this study, our results indicated the decreased exercise ability, declined memory, and impaired learning induced by HIE, while these alters could be partly reversed by acupressure treatment. Besides, acupressure treatment involves several dominant characteristics, such as being safe, well‐tolerated, and effective, from the cognitive and motor levels.

Acupressure method, stimulating acupoints and meridians with fingers, could lead to acupuncture‐like effects [35], also related with the selectivity of spots, duration, and dose of stimulation [36]. BL23 is the associated Shu point of the kidney [37]. According to the theory of Chinese medicine, BL23 can not only promote blood activity but also excel in making vitality and energy elevated [38, 39]. Patients with HIE diseases often manifested deficiency and stasis, accompanied by motor disturbance, cognitive damage, and even limb disownership. Therefore, the acupoints of BL23 were selected in the study to treat the HIE model rats. BL23 is located in the bladder meridian, on the lumbar and spine areas [40]. In accordance with previous studies [41], our results suggested that stimulating BL23 continuously has promising results in motor and cognitive impairment. Acupressure is a noninvasive and cost‐effective method. However, few studies elucidated the underlying molecular mechanisms of synaptogenesis based on acupressure involved in HIE treatment. At the macro level, our study found that HIE caused atrophy of brain tissue and slow growth, with ongoing secondary behavioral and cognitive changes, whereas acupressure reversed these phenomena. In the molecular biological detection, acupressure upregulated the expression of synaptic plasticity–related signaling molecules such as NMDAR and AMPAR. Intriguingly, cognitive function and neuroplasticity were interrelated, with reduced neuroplasticity possibly playing a key role in the impaired cognitive change.

Glutamate receptors play a central role in synaptic plasticity and cognitive function [42]. NMDAR and AMPAR are the main glutamate receptors in the central nervous system, both of which have been shown to play an important role in learning and memory in the brain. Specifically, NMDAR, which is a key mediator of intracellular calcium influx and synaptic LTP [43], and AMPAR, which regulates rapid excitatory synaptic transmission, together determine the efficacy of synaptic plasticity. NMDAR is highly permeable to calcium ions and participate in synaptic plasticity, synaptic excitatory conduction, and excitotoxicity [44]. The AMPAR is responsible for the depolarization of the postsynaptic region, known as the excitatory postsynaptic potential (EPSP) [45]. However, in the case of HIE, weakened receptor function may disrupt glutamate signaling, resulting in excessive release of glutamate, leading to excitotoxicity, weakening synaptic strength, and ultimately resulting in cognitive dysfunction [46]. The findings of this study are consistent with previous studies, which showed that HIE can lead to significant impairment of learning‐ and memory‐related behaviors in rats, which is closely related to the downregulation of NMDAR and AMPAR on the postsynaptic membrane of the hippocampus.

In the nervous system, neurons are the basic structural and fundamental units [47], helping convey information and control motion. Furthermore, nerve cells also participate in cognitive regulation, especially neurons in the hippocampus [48]. The efficient functional connectivity between neurons depends on the LTP inhibition to altering signal intensity, a process termed synaptic plasticity [49]. The neuron activity is in a state of dynamic change, favoring synaptic information across [50]. Previous studies have illustrated that enhancing neurogenesis is efficacious in alleviating HIE‐caused cerebral palsy [51]. Similarly, in the study, we found that neuronal damage occurred after HIE in the hippocampus of rats, specifically accompanied by synaptic cleft narrowed, synaptic membranes thinned, dendritic spike reduction, and dendritic length decreased. Moreover, the morphologies of neurons were stained deeply, arranged disorderly, and atrophied significantly. Interestingly, acupressure treatment could enhance neuronal connections within the hippocampus, which provides a new perspective on acupressure applications in HIE‐caused cerebral palsy treatment.

In the field of neuroscience, as a macroscopic electrical signal reflecting neural network population activity, the LFP essentially represents extracellular recordings of the summated currents in the dendrites of local pyramidal neurons [52]. This electrical fluctuation primarily originates from the integration of postsynaptic potentials mediated by AMPARs and NMDARs on the postsynaptic membrane [53]. Notably, the amplitude characteristics of LFP are closely associated with the functional status and density of glutamate receptors within the PSD, particularly the calcium influx mediated by NMDARs, which plays a critical role in regulating LFP dynamics [54]. This study, through precise localization of the pyramidal cell layer in the CA1 region of the rat hippocampus and employing EEG recording techniques under awake, freely moving conditions, systematically revealed the profound impact of HIE on glutamatergic synaptic transmission. Experimental data demonstrated characteristic biphasic waveform distortions in the CA1 LFP of HIE‐caused rats: a significant reduction in the slope of early fast EPSPs, indicating decreased efficiency of AMPAR‐mediated synaptic responses, and a shortened duration of the late NMDAR‐dependent slow component, suggesting impaired NMDA receptor channel opening probability or calcium permeability. These findings complement previous research to form a coherent evidence chain. For instance, Sweda et al. [55] confirmed that HIE reduces glutamate release at mossy fiber terminals in the hippocampus. It is crucial to note that our findings in awake animal models more accurately reflect the true pathological features of synaptic dysfunction compared to traditional anesthetized state recordings—anesthetic agents’ suppression of NMDAR function may obscure potential compensatory mechanisms [56].

Since synaptic activity occurs, AMPAR and NMDAR are activated, accompanied by extensive glutamate release [57]. The Ca^2+^ influx of NMDAR activates calmodulin‐dependent protein kinase (CaMK), which phosphorylates downstream targets such as the subunits of AMPAR to increase their conductivity [58]. As a scaffold protein, PSD95 may aggregate NMDAR and other signaling molecules to promote signaling [59]. Concomitantly, SYP regulates the release of neurotransmitters at the presynaptic level and may affect the strength of the postsynaptic signal, such as by regulating the amount of glutamate released, thus affecting the induction of LTP [60]. It has been found that, in HIE, excessive activation of NMDAR leads to Ca^2+^ overload, activates CaMK, and promotes PSD95 aggregation, which in turn destroys the balance between AMPAR/NMDAR and ultimately exacerbates synaptic dysfunction and neuronal death [61]. In this study, we detected the expression of both AMPAR and NMDAR in the hippocampus. It is indicated that both the protein levels of AMPAR and NMDAR were decreased in the HIE control group, while these results can be partially eliminated by acupressure intervention. These molecular changes were consistent with prior morphological results stained by Golgi and immunohistochemistry.

In the consideration of setting up a group for pressing fake acupoints, previous studies have found that “non‐acupoints” are the most commonly chosen. However, fake acupoints are also a form of skin‐based touch therapy and can have therapeutic effects. Any form of acupoint stimulation can be considered an emotion‐centered therapy [62]. Therefore, this experiment adopted a fake surgery group. Unlike the HIE control group and the acupressure group, this group did not experience ischemia or hypoxia and did not undergo any intervention, serving as a routine control to verify the success of the model and the effectiveness of acupressure.

Overall, HIE‐induced rats can be used as a reliable animal model of HIE. Acupressure is effective and safe in improving motor and cognitive behavior, increasing the number of neurons, extending dendrite length, increasing the density of spiny structures, promoting glial fibrils, and alleviating the rate of synaptic abnormalities in HIE rats. Importantly, acupressure treatment can upregulate the expression of proteins associated with neuroplasticity in the hippocampus, including AMPAR and NMDAR. Our study provides a new perspective on the approach to ischemia‐hypoxia cerebral palsy and elucidates its associated molecular mechanisms. Further studies are necessary to elucidate these mechanisms.

## 5. Conclusion

This study found that acupressure exerted therapeutic effects in a rat model of HIE‐induced cerebral palsy. Acupressure at BL23 inhibited the hyperactivity of AMPAR/NMDAR, which was associated with improved hippocampal neuroplasticity in HIE rats. These changes may be related to increases in the number of neuronal dendritic spines and the expression levels of related proteins.

## Author Contributions

Xinghe Zhang and Xiantao Tai were responsible for the study concept and design. Yiping Chen and Yongli Li conducted the animal experiment and wrote the manuscript. Yong Liao conducted the experiments and collected the data. Chenggui Xu helped to analyze the data.

## Funding

The study was supported by grants from the National Natural Science Foundation of China (Grant 82360980), the Applied Basic Research Project of Yunnan Education Department (Grants 2024Y388 and 2025Y0643), and the Yunnan Province Innovation Team of Prevention and Treatment for Brain Diseases with Acupuncture and Tuina (Grant 202405AS350007).

## Disclosure

All authors have approved the final version of the manuscript.

## Ethics Statement

In this study, all rat care and experimental procedures were approved by the Animal Ethics Committee of Yunnan University of Chinese medicine (the Ethics Approval Number R‐062023G004) and implemented in accordance with the National Institutes of Health Guide lines for the Nursing and Use of Laboratory Animals (Animal Qualified Certificate Number 110324241102605772; Animal Production License Number SCXK [JING] 2024‐0001; Experimental Animal Use License Number SYXK [DIAN]K2022‐0004).

## Consent

Consent is not applicable. The manuscript does not include details, images, or videos relating to individual participants.

## Conflicts of Interest

The authors declare no conflicts of interest.

## Data Availability

The data that support the results of this study are available upon request from the corresponding author, Xinghe Zhang.
